# Behind Resveratrol Stabilization by Carboxymethylated (1,3/1,6)-β-d-Glucan: Does the Polyphenol Play a Role in Polymer Structural Organization?

**DOI:** 10.3390/ijms18092006

**Published:** 2017-09-19

**Authors:** Antonio Francioso, Simone Dinarelli, Marco Girasole, Laura Cervoni, Maria d’Erme, Francesco Mura, Alberto Boffi, Elita Montanari, Luciana Mosca

**Affiliations:** 1 Department of Biochemical Sciences “A. Rossi Fanelli”, Sapienza University of Rome, 00185 Rome, Italy; Laura.cervoni@uniroma1.it (L.C.); Maria.derme@uniroma1.it (M.d’E.); Alberto.boffi@uniroma1.it (A.B.); luciana.mosca@uniroma1.it (L.M.); 2 ISM Institute of Material Structure, CNR National Research Council-Rome, 00185 Rome, Italy; simone.dinarelli@ism.cnr.it (S.D.); Marco.girasole@artov.ism.cnr.it (M.G.); 3 CNIS Research center for Nanotechnology Applications-Rome, 00185 Rome, Italy; francesco.mura@uniroma1.it; 4 Department of Drug Chemistry and Technology, Sapienza University of Rome, 00185 Rome, Italy; elita.montanari@uniroma1.it

**Keywords:** CM-glucan, resveratrol, supramolecular chemistry, spectroscopy, calorimetry, AFM, SEM

## Abstract

Resveratrol stability in solution can be improved by combining the polyphenol with carboxymethylated (1,3/1,6)-β-d-glucan (CM-glucan), a carbohydrate polymer widely used in the food and pharmaceutical industries. The present work was undertaken to elucidate the mechanism behind this stabilizing effect. The supramolecular structural, physico-chemical and morphological features of the CM-glucan/resveratrol complex have been studied under different physical and chemical stimuli by means of spectroscopic techniques, microscopy and physical methods such as UV-Visible spectroscopy (UV-Vis), spectrofluorimetry, Circular Dichroism (CD), Infrared spectroscopy (FT-IR), Differential Scanning Calorimetry (DSC), Atomic Force Microscopy (AFM) and Scanning Electron Microscopy (SEM). Our experimental data indicate that CM-glucan conformational organized architecture in aqueous solution is enhanced in the presence of resveratrol, suggesting that the polyphenol is able to confer a high degree of order to the polymer by a probable cooperative structural organization that results in a long term stabilization for the polyphenol.

## 1. Introduction

**Figure 1:**
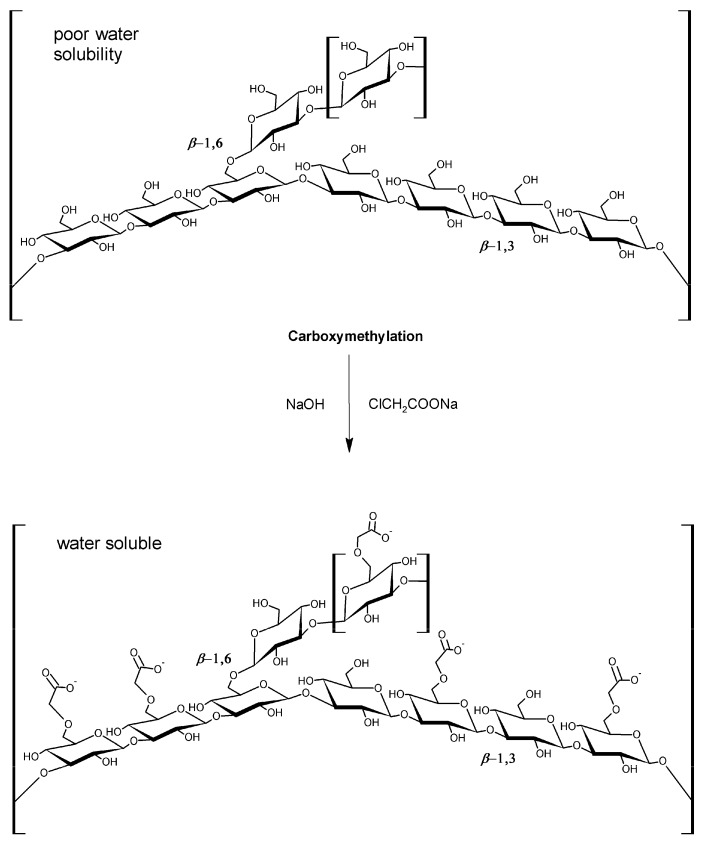
Structure of (1,3/1,6)-β-d-glucan and its carboxymethylated derivative (CM-glucan).

β-Glucans are non-cellulosic polymers of β-d-glucose belonging to a group of biologically active biopolymers which exist in different structural organizations [1,2,3,4], i.e. linear with glycosidic bonds in the β(1→3) position (i.e., curdlan) or branch-on-branch or cyclic structures (1→3,1→6)-β-glucans. They are widespread in nature such as in fungi, cereals, bacteria or seaweeds [5,6,7,8]. The unique properties of β-glucans have led to several applications including the formulation of food gels or application as helical scaffolds for nanostructure formation [1,2]. The reason why so much attention has been devoted to β-glucans is that they are a typical example of biological response modifiers with pronounced immunomodulating activity which confer them anticancer, antioxidant, antiviral and wound healing properties [9,10]. These biopolymers are also widely employed as nano-carriers for the delivery of active compounds (e.g., hydrophobic drugs which show low bioavailability in the human tissues) [11,12]. However, due to their poor water solubility, β-glucans show limited applications for aqueous formulations; therefore, several attempts for improving their hydrophilicity have been made. Among them, carboxymethylation is one of the most common structural modifications. It makes β-glucans more soluble in water compared to the native polymer (Figure 1) [13], allowing the retention of such important β-glucans properties (e.g., anticancer and antioxidant properties) [14,15].

**Figure 2:**
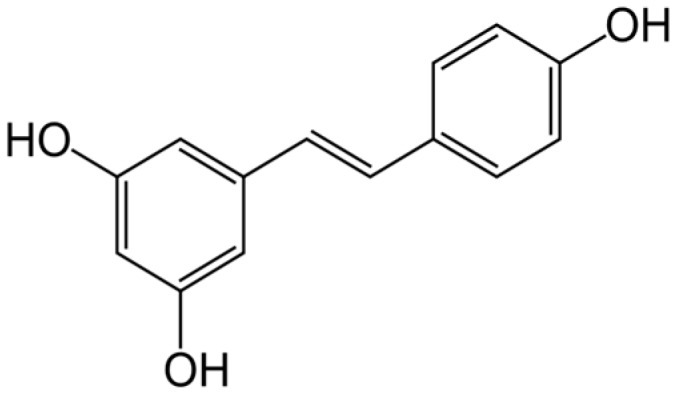
Structure of trans-Resveratrol.

One of the most attractive properties of β-glucan is its structure-forming ability which, combined with its pharmacological activity, makes this compound interesting for the formulation of pharmaceutical preparations [9,16,17,18]. Exploiting these multiple properties, our group has recently developed an aqueous formulation by combining carboxymethylated (1,3/1,6)-β-d-glucan (CM-glucan) with resveratrol, a non-flavonoid polyphenolic compound abundant in grapes, peanuts and other foods that are usually consumed as part of the human diet [19] (Figure 2).

Resveratrol is well known for its antioxidant, anti-inflammatory, antiviral, cardioprotective, neuroprotective, chemo-preventive, and anti-aging properties [20]. However, its poor solubility, low stability in aqueous environment and low bioavailability limit its use in the pharmaceutical field. To overcome these drawbacks, CM-glucan was employed for improving resveratrol water solubility. In this respect, in a previous work, we demonstrated that CM-glucan is able to dramatically improve long-term stability of resveratrol in aqueous media [19].

Therefore, in the present work, CM-glucan/resveratrol complex was studied with the aim to investigate its structural, physico-chemical and morphological properties using several spectroscopic and imaging techniques such as Circular Dichroism (CD), Differential Scanning Calorimetry (DSC), UV-Visible spectroscopy (UV-Vis), spectrofluorimetry, infrared spectroscopy, Atomic Force Microscopy (AFM) and Scanning Electron Microscopy (SEM) [21]. These techniques allowed the direct visualization of the structural organization of CM-glucan in response to resveratrol addition.

## 2. Results and Discussion

### 2.1. Solubility of Resveratrol in CM-Glucan Aqueous Solution

**Figure 3:**
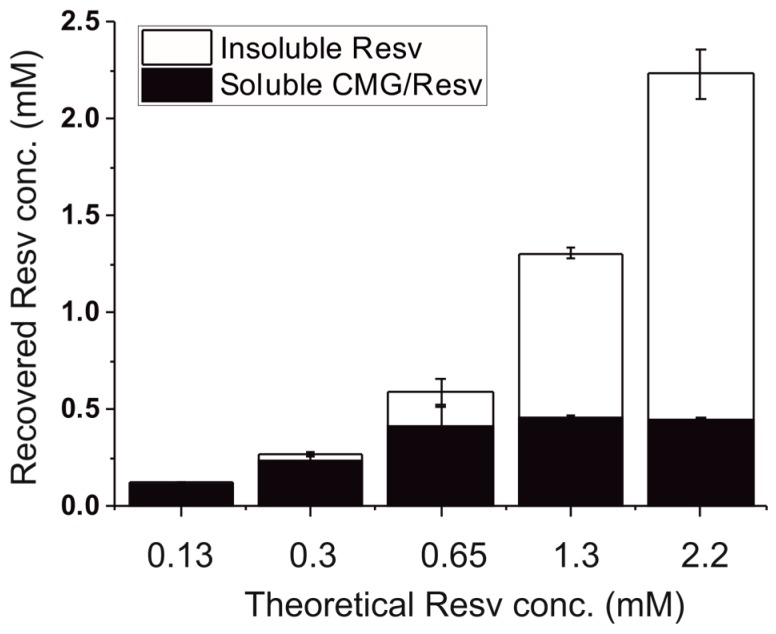
Solubility of resveratrol in aqueous CM-glucan (CMG) solutions using several resveratrol concentrations (0.13, 0.3, 0.65, 1.3 or 2.2 mM) and 1 mg/mL CM-glucan in water. Samples were sonicated for 15 min at 25 °C and then vortexed and centrifuged at 16,000× *g*, for 15 min at 25 °C. The absorbance of the supernatant was measured at 306 nm. The insoluble fraction was recovered and total resveratrol amount was measured.

Resveratrol is a polyphenol that shows many attractive biological properties, however, its poor solubility in aqueous environment limits its applications in the pharmaceutical field. To overcome this drawback, CM-glucan was employed to improve resveratrol water solubility. Different amounts of resveratrol were added to a fixed concentration of CM-glucan aqueous solution and after centrifugation, resveratrol concentration was determined in the supernatant. As shown in Figure 3, the concentration of resveratrol in the CM-glucan aqueous matrix increased reaching a plateau at 0.45 mM, which is about three times higher with respect to its solubility in water (~0.14 mM). It should be pointed out that 0.30 mM was the maximal resveratrol aqueous concentration obtained up to now, without using any emulsifying agents [22,23]. Our results demonstrate that CM-glucan confers to resveratrol a high degree of solubility in water. This result confirms the ability of glucans to act as carriers for lipophilic molecules, as already demonstrated for curdlan which efficiently encapsulated epirubicin making it more active as anticancer drug [9,12,24].

### 2.2. Spectroscopic Studies of CM-Glucan/Resveratrol Complex

**Figure 4:**
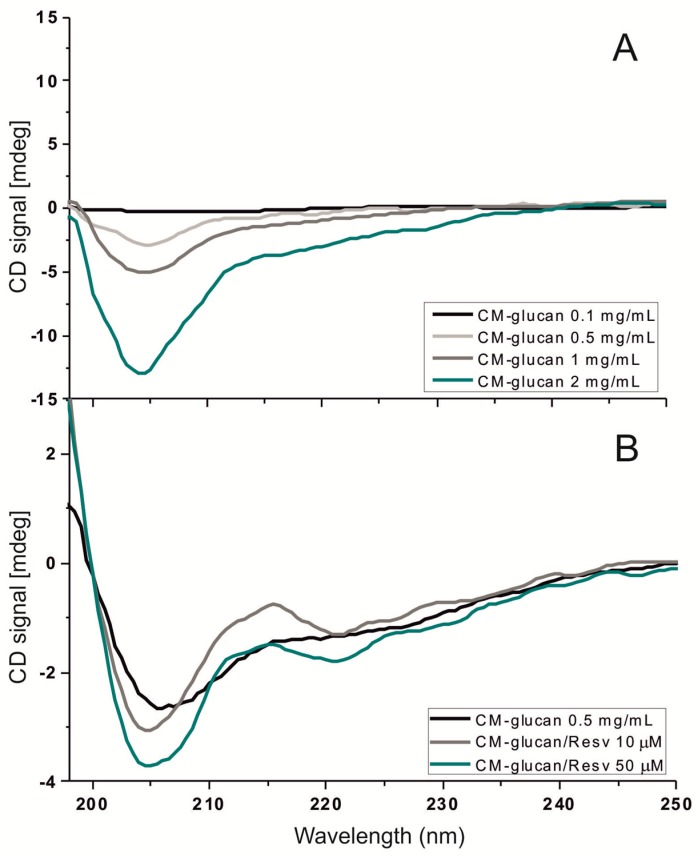
(**A**) Circular Dichroism (CD) spectra of CM-glucan at several concentrations (0.1–2 mg/mL) in water; and (**B**) CD spectra of CM-glucan in the presence of increasing resveratrol concentrations (10–50 µM). Analyses were performed at 25 °C and scanned from 196 to 250 nm.

The evidence that CM-glucan maintains resveratrol in solution, improving its solubility, prompted us to study CM-glucan/resveratrol complex from the physico-chemical and conformational point of view. Several authors have demonstrated the ability of (1,3/1,6) β-glucans and derivatives to give rise to ordinate secondary structures [25,26,27]. These authors suggest that β-glucans having an average degree of polymerization number (DPn) above ~200 show ordered conformations. To evaluate the effects of resveratrol on the CM-glucan conformation, the dichroic behavior of carboxylic group of CM-glucan was analyzed in the presence or absence of resveratrol. Indeed, the –COOH group allows directly evidencing the presence of dissymmetrical active structures in the polymer. Circular Dichroism (CD) spectra were recorded at increasing concentrations of CM-glucan in water, evidencing an intense proportional CD signal in the –COOH absorption region (200–220 nm) (Figure 4A). When resveratrol was added to the CM-glucan solution, the CD signal at 205 nm increased proportionally to resveratrol concentration, suggesting the ability of the polyphenol to augment the degree of dissymmetrical organization of CM-glucan (Figure 4B).

**Figure 5:**
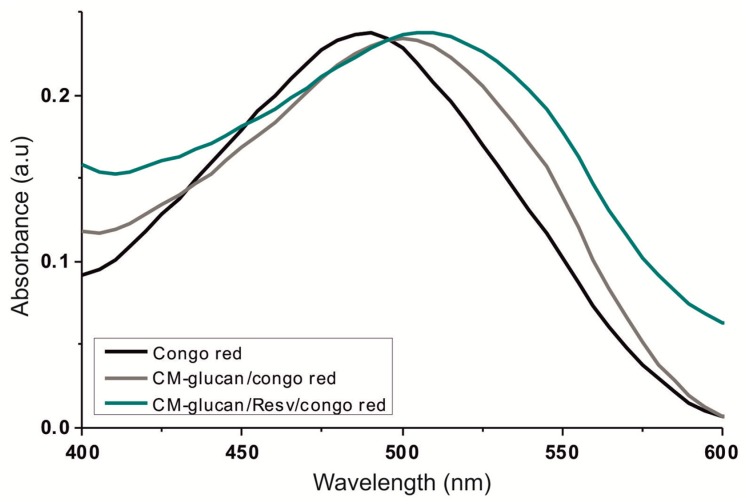
Congo Red binding assay. Congo Red UV-Visible spectroscopy (UV-Vis)absorbance was checked from 400 to 600 nm at 25 °C. Black line represents the control of Congo Red alone (10 µM); the grey line corresponds to the mixture of CM-glucan/Congo Red (1 mg/mL and 10 µM, respectively); the green line represents the complex CM-glucan/Resv in the presence of Congo Red (1 mg/mL, 200 µM, and 10 µM, respectively).

Polysaccharides existing in an ordered conformation form a complex with Congo Red dye in dilute alkaline solution, thanks to the ability of 1→3 glucans to interact with the planar structure of the dye. These interactions cause a change in the spectroscopic properties of Congo Red resulting in a shift of the UV-Vis λ_max_ [27]. In this respect, as shown in Figure 5, when CM-glucan was added to Congo Red in dilute NaOH solutions, a bathochromic shift of 15 nm was observed, indicating the presence of a secondary structure able to bind and form a complex with Congo Red. When resveratrol was added to this solution, the bathochromic shift further increased, reaching a maximum of 20 nm at 200 µM resveratrol. This additional shift in dye absorbance may indicate that resveratrol further reinforces the interaction between Congo Red and CM-glucan, probably conferring a further degree of order to the polymer. These data are in agreement with our experiments on the dichroic behavior of CM-glucan, in which the CD signal increases in the presence of resveratrol, suggesting that this polyphenol causes an enhancement of the polymer dissymmetrical regular structures.

### 2.3. Thermal Transitions of CM-Glucan

From UV-Vis spectroscopic data, we found that the carboxymethylated water soluble CM-glucan shows a certain degree of secondary structure organization in water solutions. Surprisingly, when CM-glucan is associated to resveratrol the binding of Congo Red dye is stronger, indicating that polyphenol promotes the formation of ordinate structures that can bind and coordinate better Congo Red molecules. In particular, the bathocromic shift of the dye is related to polymer helix-coil-helix transition [27]. These data are in agreement with circular dichroism experiments in which the presence of resveratrol gives rise to a more intense CD signal of the polymer. The CD signal of CM-glucan indicates the presence of dissymmetrical regular structures that increase in a proportional manner with resveratrol concentration.

These results drove us to deeply investigate this kind of transition, in particular by the aid of calorimetry technique. It has been reported that 1,3-β-d-glucans are able to form triple-strand helical structures and some of them, such as schizophyllan, can form complexes with polynucleotides, carbon nanotubes, and some hydrophobic polymers that can be studied by DSC analysis [28,29,30].

**Figure 6:**
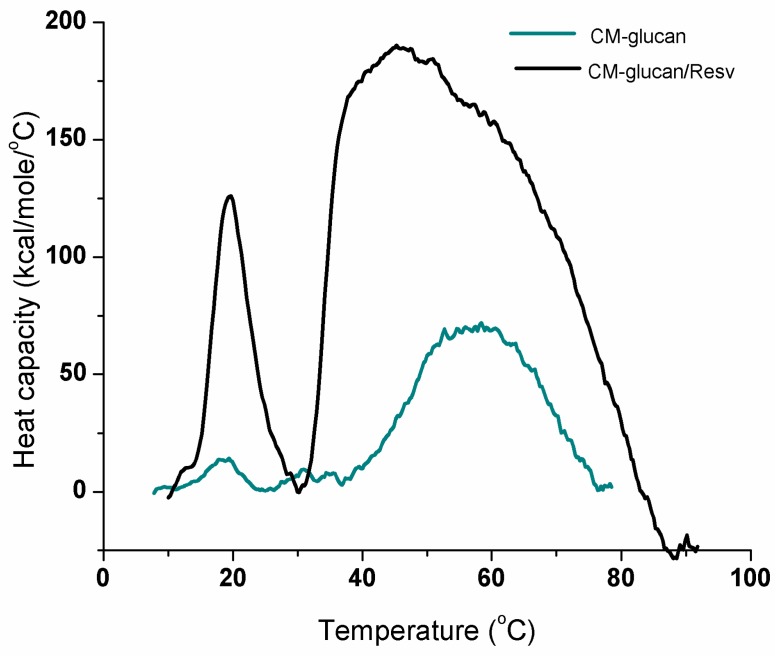
Differential Scanning Calorimetry (DSC) curves of 0.1 mg/mL CM-glucan in aqueous solution in the presence or absence of 200 µM resveratrol. First transition at 19 °C corresponds to water associated triple-stranded helical chain of the polymer and the second broad peak between 40 and 80 °C is referred to intra- and intermolecular hydrogen bonds involving the “water-free” state of the polymer.

Figure 6 shows DSC curves of CM-glucan with or without resveratrol in aqueous solution. The thermal transitions in the range from 10 to 80 °C correspond to the different conformational states of CM-glucan in aqueous environment. The first transition *T*_m1_ (temperature of the maximum heat capacity) at 19 °C corresponds to a change in the water associated triple-stranded helical chain of the polymer, a process which needs of a relatively low energy. In this region, the hydrogen bond interactions between the side-chain glucose residues and the surrounding water clusters maintain a well-organized structure of the glucan triple helix. CM-glucan needs of a slightly higher energy to achieve this transition (from triple helix I to triple helix II “water free” conformation) [31], which is due to the new hydrogen bonds that –COOH forms with the water stabilized network.

When resveratrol is added, a significant enhancement of the peak at 19 °C can be observed, suggesting that resveratrol is able to confer a high degree of order to the polymer in terms of CM-glucan water associated triple-stranded helical chains. Table 1 reports the *T*_m_ (temperature of the maximum heat capacity), ΔHc (calorimetric enthalpy of denaturation), and ΔHvH (van’t Hoff enthalpy of denaturation, equal to ΔHc for a two-state transition), obtained by deconvolution.

The second broad peak from 40 to 80 °C corresponds to the transitions of the various ordinate secondary CM-glucan structures in which the conformations are stabilized by intra- and intermolecular hydrogen bonds involving the “water-free” state of the polymer [32,31]. Resveratrol has a dramatic impact on these transitions. Indeed, we observed that, in the presence of resveratrol, the intra- and intermolecular water free thermal transitions of the polymer increases. This latter phenomenon could be attributed to the dissociation of the polyphenol from the polymer skeleton.

### 2.4. Infrared Analyses

**Figure 7:**
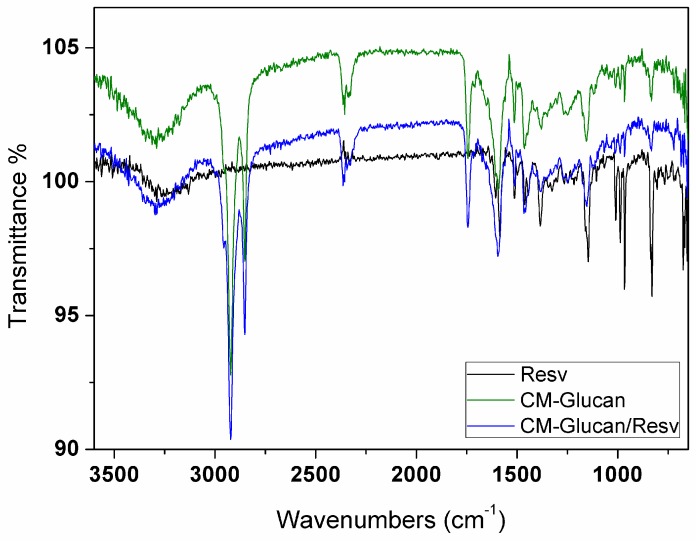
Infrared spectra of dried resveratrol (black line), CM-glucan (green line) and their complex (blue line). Analyses were performed at 25 °C from 3600 to 700 cm^−1^.

As shown in Figure 7, infrared spectra of CM-glucan are in agreement with previous studies [13,19]. However, in the present spectra, some differences are present, due to the lack of solvent related signals in the solid state. Interestingly, Infrared spectroscopy (FT-IR) spectrum of CM-glucan/resveratrol complex shows a marked decrease of resveratrol phenolic C–O stretches signals (1010, 988, 966 cm^−1^). This effect could be ascribed to a change in the chemistry of phenolic moiety and hence to a change of the C–O dipolar moment. This latter phenomenon could be due to the deprotonation of the OH residues that, thanks to the stabilization of the anion by electron delocalization, leads to the formation of a phenolate. In this case, the C–O linkage could assume a partial double bond character. This phenomenon can partially explain the broadening of the signal at 1600 cm⁻^1^, in which both the C=O signals of the carboxyl anion for glucan and the phenolate signal of resveratrol anion are overlapped.

### 2.5. Fluorescence and Binding Studies

**Figure 8:**
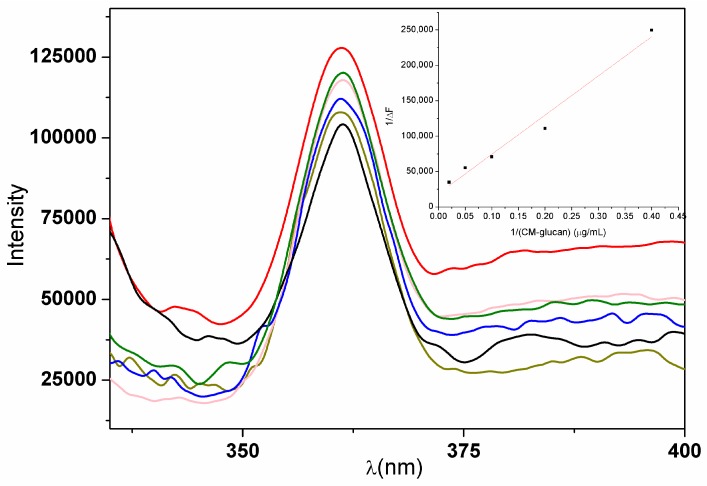
Fluorescence spectra of resveratrol in the presence of increasing CM-glucan concentrations (0, 2.5, 5, 10, 20, and 50 µg/mL, are represented by the black, dark, green, blue, pink, light green and red lines, respectively).

Fluorescence studies on resveratrol and CM-glucan complex allow to investigate the theoretical binding constants and to elucidate in more detail the nature of the chemical interactions between the two compounds. In general, a λ_max_ shift to shorter wavelengths in the emission spectrum is observed for organic molecules as the polarity of the environment decreases, whereas an increase in fluorescence intensity is representative of a stable binding of the molecule with an interactor [33]. Generally, these two phenomena are concurrent. Figure 8 shows the increase in fluorescence intensity of resveratrol in aqueous solutions at increasing concentrations of CM-glucan. As shown from the graph, a linear proportional increase is observed from 0 to 50 μg/mL of CM-glucan at 10 μM resveratrol. Previous studies have shown that polyphenols tend toward self-association in solution as a result of hydrophobic stacking of aromatic phenolic rings [34,35]. Resveratrol possibly causes self-association at concentrations higher than 40 μM, which results in polyphenol fluorescence self-quenching. Maintaining resveratrol at 10 μM and increasing CM-glucan concentrations allows studying the association rate in absence of self-association quenching effects.

In our experimental conditions, emission λ_max_ of 10 μM resveratrol in aqueous solution is at 361 nm when excited at 320 nm. Notably, when CM-glucan is present in the solution no shift in resveratrol emission λ_max_ is observed. This phenomenon is a remarkable index of the nature of the interactions between the two molecules. Indeed, the increase in emission intensity of CM-glucan/Resv complex is not accompanied by a blue shift, thus indicating that the binding of the polyphenol occurs probably with the hydrophilic moieties of the polymer and not with a lipophilic pocket or region, as for example is observed for many protein binding sites [35]. These data are in agreement with infrared spectroscopic data, and strongly support the hypothesis that the binding between CM-glucan and resveratrol occurs via hydrogen bonding between fenolic hydroxyls of the polyphenol and the carboxylic and hydroxyl moieties of the polymer, and not through hydrophobic interactions with the carbon backbone.

As previously demonstrated, several molecules such as Congo Red, Calcofluor and other stilbene-related dyes can bind glucan polysaccharides due to their polar and stereochemical affinity with these polymers [26,36,37]. By combining fluorimetric, infrared, DSC, and Congo Red binding data, it is reasonable to hypothesize that the interaction of CM-glucan with resveratrol is the result of both polar interaction sites and stereochemical stilbene-like conformation which give rise to an optimal binding. Thus resveratrol, being a hydroxylated stilbene, has the advantage of a completely planar structure that probably can be coordinated by the terminal phenolic moieties. This hypothesis is also strengthened by the evidence that non-stilbenic phenolics alone are not sufficient to give rise to the same interactions that are shown with resveratrol.

Generally, complexes involving non covalent bonds are reversible. In our experimental conditions, the binding constants (K_a_) for resveratrol fluorescence data can be analyzed by the following equation [38]:

(1) $$\frac{1}{\Delta F}=\frac{1}{{\Delta F}_{\max}}+\frac{1}{K_{a}{\text{×}\Delta F}_{\max}\text{×}(\mathrm{CM}-\mathrm{glucan})}$$

where ΔF is the difference in fluorescence value at 361 nm in the presence or absence of CM-glucan increasing concentrations; ΔF_max_ is the maximal fluorescence intensity change; K_a_ is the binding constant; and (CM-glucan) is the polymer concentration. Double reciprocal linear plot of 1/ΔF and 1/(CM-glucan) according to the equation is given as inset in Figure 8. K_a_ binding constant can be calculated from the intercept and slope of the graph line.

Being CM-glucan a heterogeneous mixture of polymers with different molecular weights (in the range 250 to 550 kDa), K_a_ values were calculated for the maximum, the minimum and the average molecular weight (about 400 kDa) of the polysaccharide and are, respectively, 0.62, 1.37 and 0.86 × 10^10^ M^−1^. Constant binding in the sub-nanomolar range is in agreement with previous studies in which Congo Red and stilbenoid dyes were investigated for their capacity to bind glucans, proposing that each molecule of Congo Red can bind six d-glucose residues of the glucan chain [25,39].

### 2.6. Morphological Studies of CM-Glucan/Resveratrol Complex

From a morphological point of view, glucans in water adopt a rigid triple-helix conformation that can be altered by a denaturation–renaturation cycle through several physical and chemical modifications [40]. At a very low concentration, different structures have been observed after renaturation; the most commonly reported are the circular (observed for different glucan derivatives) and the rod-like one (observed for several glucans) [41,42,43].

AFM has been previously used for characterizing compounds with a structure similar to CM-glucan, such as scleroglucan and different oat β-glucan isolates [44,45]. The other microscopy technique employed in this paper, SEM, is suitable to provide a rapid and direct high-resolution visualization of the sample’s morphology; moreover, if equipped with an Energy Dispersive X-Ray (EDX) spectrometer, it can provide a quantitative elemental composition map of the sample. This technique has been already employed in the study of both rod-like [44] and circular structures of different glucan derivatives, after a thermally induced denaturation and renaturation process. Hence, AFM and SEM can be combined together to achieve an in-depth characterization of the sample structures, in particular, to study highly structured surfaces such as those obtained by deposition of highly concentrate glucan solutions. Indeed, widely different morphological landscapes have been observed for highly concentrated solutions of a (1,3/1,6)-β-d-glucan extracted from yeast [46]. When air-dried films of this compound were created, the triple-helix, rod-like and circular structures were converted into macromolecular aggregates of different shapes, ranging from a branched tree-like structure to granules [40].

**Figure 9:**
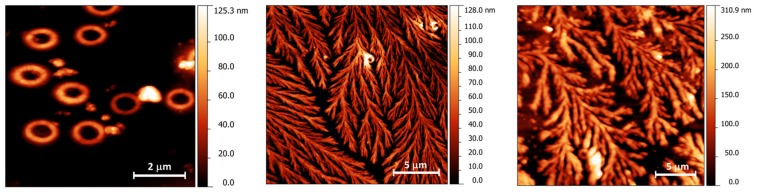
Atomic Force Microscopy (AFM) images of CM-glucan: in water, pH 6.5 (**left**); in alkaline solution, pH 11 (**middle**); and in association with resveratrol in water, pH 6.5 (**right**).

It is well known that 1,3-β-d-glucan can form triple-strand helical structures and that this ordinate structure is favorite in slightly alkaline solutions. AFM results show the “morphological face”, in the microscopic scale, of the fractal-like state of CM-glucan dissolved in alkaline solution (Figure 9, central panel) and the ring-like structure of about 2 µm in diameter observed at pH 6.5 (Figure 9, left panel). Surprisingly, resveratrol behaves like a mimic of the “ordering glucan factor” nearby neutral pH conditions (Figure 9, right). The presence of resveratrol confers to CM-glucan a branched structure similar to that shown for CM-glucan in alkaline solutions. Notably, dynamic light scattering (DLS) analysis confirmed the presence of CM-glucan structures (2–4 µm in diameter) at pH = 6.5; in contrast, at pH = 11 as well as after the addition of resveratrol, the polydispersity index dramatically increased, making these samples not suitable for DLS results.

**Figure 10:**
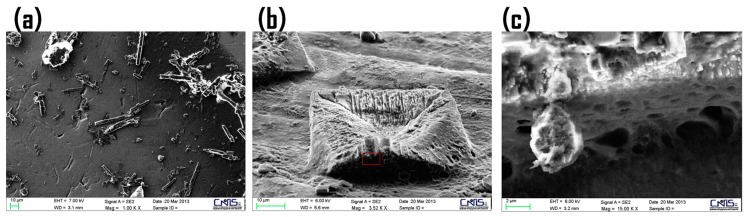
SEM images of: resveratrol/CM-glucan complex freshly prepared (**a**); after 12 months of storage at room temperature (**b**); and zoom of the aged sample (red rectangle) (**c**).

Our previous work evaluating the long term stability of a CM-glucan/Resv formulation indicate that after a slight decrease in the first two months, the polyphenol is stable up to 18 months in aqueous solution at room temperature. With the aim to investigate the CM-glucan/Resv complex structure over the time we performed SEM analyses on both freshly prepared samples and on samples stored for 12 months (Figure 10). The analyses clearly reveal that the morphology of the long-term stored sample is significantly different from the freshly prepared one, showing the presence of ordered superstructures with geometrically precise organization, with a dimension of about 50 μm (Figure 10a,b). Figure 10c shows the zoom of the lateral area of one of those superstructures, evidencing the presence of a sponge-like matrix on the surface of the aged sample.

This phenomenon could be due to the time-dependent entrapment of the insoluble resveratrol into the polymer matrix. In this case, the super-ordered structure could act as a reservoir for resveratrol, releasing soluble fractions of the drug when the soluble yet present fraction are gradually degraded during long-term storage. In this case, the aqueous pocket of CM-glucan net matrix could accommodate the soluble reservoir fraction of resveratrol.

**Figure 11:**
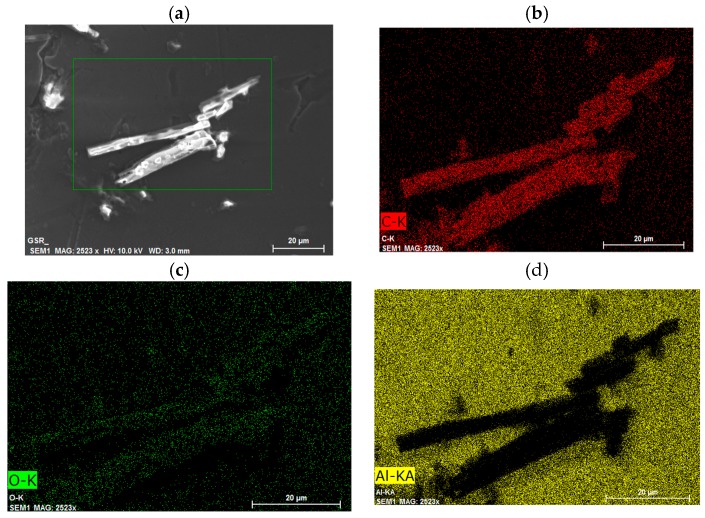
(**a**) SEM image; and (**b**–**d**) Microanalyses of carbon (red), oxygen (green) and aluminum (yellow), respectively, of CM-glucan/resveratrol (freshly prepared formulation).

Figure 11 performed reports the microanalyses on one of the fresh sample reported in Figure 10a. The figure shows the co-localization of the two organic molecules containing Carbon and Oxygen elements (Figure 11b,c, respectively) as well as (in yellow) (Figure 11d) the silicon wafer with aluminum surface. This result remarks from a macroscopic morphological point of view that the two chemical entities strictly interact and co-localize in the same physical space.

### 2.7. Cell Viability of Resveratrol/CM-Glucan Association

**Figure 12:**
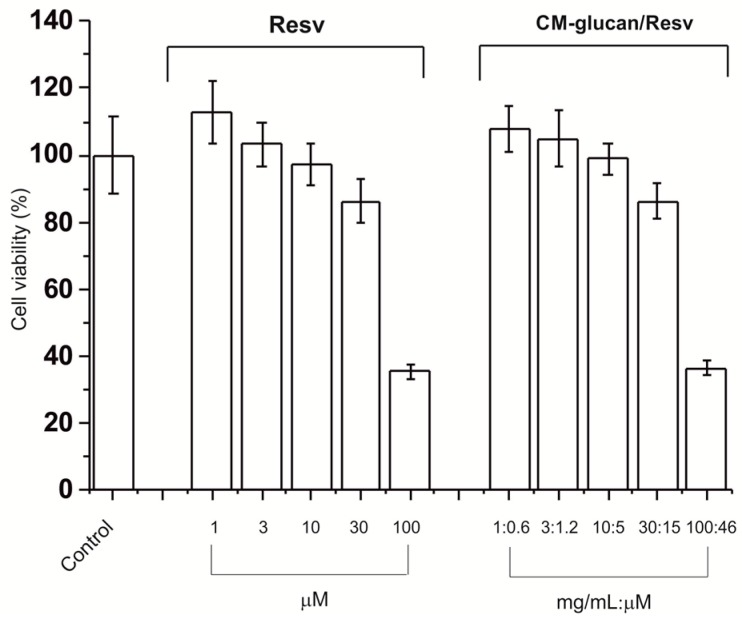
Viability of HaCaT cells line incubated for 48 h at 37 °C with resveratrol, CM-glucan, or the CM-glucan/Resv complex. Control is referred to the untreated cells and the error bars represent the standard deviation over three samples. Cell viability (%) values were normalized against the control.

Figure 12 shows the viability of HaCaT cells treated with several resveratrol concentrations (1–100 µM) and incubated for 48 h. The results demonstrate that resveratrol has negligible toxicity at concentrations ≤ 30 µM; conversely, it caused a significant reduction in cell viability at 100 µM.

One micromolar concentration evidences a slight proliferative effect meanwhile increasing concentrations show a proportional anti-proliferative effect from 1 to 100 μM. Moreover, CM-glucan did not affect the cell viability at the tested concentrations.

As shown in Figure 12, the same resveratrol concentrations tested in association with increasing proportional CM-glucan concentrations (2:1 *w/w* with resveratrol) neither protect nor negatively affect the biological effect of the polyphenol on cellular viability.

## 3. Materials and Methods

### 3.1. Solubility Studies

Several amounts of resveratrol (0.13, 0.3, 0.65, 1.3 or 2.2 mM) were dissolved in CM-glucan aqueous solution (1 mg/mL). After 15 min at 25 °C of sonication (“Selecta Ultrasons-H mod. 3000838”, JP SELECTA S.A. Laboratory equipment, Barcelona, Spain) and vortexing, samples were centrifuged at 16,000× *g* for 15 min at 25 °C. The supernatant optical density was then measured at 306 nm for determining the soluble resveratrol concentration (ε^1M^_306nm_ = 31,000), whilst the insoluble fraction (precipitate) was dissolved in Et(OH) to completely solubilize resveratrol and measure its total amount.

### 3.2. Congo Red Binding Assay

Resveratrol binding to CM-glucan was checked by monitoring the shift of Congo Red λ_max_ (10 µM in 0.01 M NaOH) in the presence or absence of 1 mg/mL CM-glucan with increasing resveratrol concentrations (from 5 to 40 µM). UV-Vis spectra were scanned from 400 to 600 nm at 25 °C with a Hitachi U-2000 spectrophotometer (Hitachi, Ltd, Tokyo, Japan).

### 3.3. Circular Dichroism (CD)

CD spectra of CM-glucan were measured at 25 °C with a Jasco J-710 automatic recording spectropolarimeter (Jasco Products Company, Oklahoma, OK, USA). CM-glucan aqueous solutions (from 0.1 to 2 mg/mL) were scanned from 196 to 250 nm to monitor the spectroscopic behavior of the –COOH moiety of the polysaccharide. The 0.5 mg/mL of CM-glucan water solution was also checked in the presence of 10 or 50 µM resveratrol.

### 3.4. Differential Scanning Calorimetry (DSC)

Heat capacity versus temperature profiles were studied with a VP-DSC differential scanning calorimeter (MicroCal. Inc., Northampton, MA, USA). CM-glucan was dissolved in water at a final concentration of 0.1 mg/mL in the presence or absence of 50 or 200 µM resveratrol. The reference cell was filled with degassed water. Both cells were kept under an excess pressure of 30 psi (200 kPa) to avoid bubbling during the scan. A scan rate of 60 °C/h was used in all the experiments. At the end of each run, the solutions were cooled and subjected to a second heating cycle under the same conditions to determine the reversibility of the transitions.

Thermograms were corrected by subtracting the instrumental base line, (obtained with both cells filled with the same solvent), and normalized to the polymer concentration. Data analysis was performed with ORIGIN 8 software (OriginLab Corporation, Northampton, MA, USA) after subtraction of a cubic base line connecting the pre- and post-transition traces. For each peak, *T*_m_ (temperature of maximum heat capacity), ΔHc (calorimetric enthalpy of denaturation), and ΔHvH (van’t Hoff enthalpy of denaturation, equal to ΔHc for a two-state transition) were obtained through a deconvolution procedure.

### 3.5. Fourier Transform Infrared Spectroscopy (FT-IR)

FT-IR measurements were performed with Nicolet Magna 760 (Thermo Scientific, Waltham, MA, USA) infrared spectrometer equipped with an ATR attenuated total reflectance device. The internal reflection element was ZnSe. Spectra were recorded at 4 cm^−1^ resolution with a DTGS detector (Deuterated Triglycine Sulfate). CM-glucan (1 mg/mL) was dissolved in water with or without 2.2 mM resveratrol. Samples were analyzed in solid state after freeze-drying.

### 3.6. Fluorescence binding Study

Fluorescence emission spectra were recorded on a SPEX-Fluoromax spectrofluorometer (Horiba Scientific, Horiba Ltd., Kyoto, Japan). Fluorescence of 10 µM resveratrol aqueous solutions was analyzed in the presence or absence of CM-glucan different concentrations (0, 2.5, 5, 10, 20, and 50 µg/mL). Emission spectra of resveratrol were recorded from 335 to 500 nm using an excitation wavelength of 320 nm with a spectral resolution of 5 nm.

### 3.7. Atomic Force Microscopy (AFM)

One-microliter drops of 1 mg/mL CM-glucan solution at pH = 6.5 (water) or 11 (0.01 M NaOH) or 1 mg/mL CM-glucan with 2.2 mM resveratrol (pH = 6.5) were deposited onto a micro cover glass (Prestige Glass, Dallas, TX, USA), previously cleaned with ethanol, and then air dried for at least 24 h at 20 °C. The AFM measurements were performed using a home-designed microscope operating in contact mode under controlled environmental condition (room temperature and constant 30% relative humidity) [47]. Silicon nitride probes (Veeco, New York, NY, USA) with asymmetric pyramidal shape and nominal tip radius of 10 nm were used. The applied vertical force was less than 1 nN and the images were collected at a scanning speed of approximately 3–4 s per row. The images were analyzed with the freeware software Gwyddion (Available online: www.gwyddion.net).

### 3.8. Scanning Electron Microscopy (SEM) and Elemental Analyses

One-microliter drops of 1 mg/mL CM-glucan water solution (pH = 6.5) with or without 2.2 mM resveratrol were cast on a silicon wafer with aluminum surface and dried at 25° C for 30 min. SEM measurements were performed using a Zeiss Auriga 405 (Carl Zeiss AG, Oberkochen, Germany) with a nominal resolution of 1 nm. The acceleration voltage was set from 1.5 to 8 keV to retain the actual morphology of the sample. The elemental analyses were assessed using an Energy Dispersive X-ray Spectrometer Bruker Quantax (Bruker Company, Billerica, MA, USA), with an energy resolution of 123 eV on the Mn Kα.

### 3.9. MTT Cell Viability Assay

HaCaT cells were maintained in DMEM high glucose supplemented with 4 mM glutamine and 1% gentamycin. Stock solutions of resveratrol (50 mM) and CM–glucan (10 mg/mL) were prepared by dissolving the compounds in dimethyl sulfoxide and water, respectively. HaCaT were treated with several resveratrol concentrations (1–100 µM) or CM-glucan (0.4–40 µg/mL) maintaining a 1:2 *w/w* ratio, respectively, between the two compounds. Cells were incubated at 37 °C in 5% CO_2_ atmosphere up to 72 h. After treatment, 20 μL of 5 mg/mL solution of 3-(4,5-dimethylthiazol-2-yl)-2,5-diphenyl tetrazolium bromide (MTT) were added in each well, and cells were incubated at 37 °C for 2 h. The supernatants were then carefully removed and the formazan crystals were dissolved in 100 μL of dimethyl sulfoxide. The absorbance was determined at 570 nm with a reference at 690 nm using an Appliskan microplate reader (Thermo Scientific, Waltham, MA, USA).

### 3.10. Statistical Analyses

Experiments were performed in triplicate and results are expressed as the mean value ± standard deviation (SD). Statistical comparison between groups was made using unpaired Student’s *t*-test (*p* < 0.05).

## 4. Conclusions

In our previous work, we demonstrated the capacity of CM-glucan to improve resveratrol stability in aqueous environment suggesting a strong interaction between these two molecules, but we did not elucidate or investigate the nature of this stabilizing effect [19].

Solubility analyses of resveratrol indicate that the polyphenol has a low tendency to self-aggregate in the CM-glucan matrix due to a possible interaction that can stabilize the molecule in a more soluble form. Taking into account the features of β(1→3) glucans, our idea was to investigate a possible interaction of resveratrol with the documented secondary structures of these polysaccharides. As reported in the literature, these carbohydrate biopolymers can form regular secondary structures such as triple helices and random coils, and this effect is more pronounced when exposed to blind alkaline environment [9,27,31,37,40]. These data indicate that resveratrol is able to induce the conformations of CM-glucan that can coordinate the water associate layer around the well-organized structural architecture of the polymer.

In conclusion, all our data concur to support the hypothesis that there is a chemical and physical interaction between the polyphenol and the polymer, and that this interaction may favor the stability of the polyphenol in the long term. Future studies will address other types of glucan polymers with different structures and degree of solubility to validate our observation and better characterize the structural modifications, which drive the conformational changes.

## Figures and Tables

**Table 1:** Differential Scanning Calorimetry (DSC) results obtained from the thermal transition of CM-glucan and CM-glucan/resveratrol complex.

Sample	*T*_m_ °C	ΔHcal kcal/mol	ΔHvH kcal/mol
CM-glucan	19.0	78.1	117.9
CM-glucan/resveratrol	19.2	409.4	123.1

Standard deviations of the fit were *T*_m_ 0.04 and ΔH 2.2.

## Abbreviations

AFM	Atomic Force Microscopy
SEM	Scanning Electron Microscopy
DSC	Differential Scanning Calorimetry
CD	Circular Dichroism
